# The Chernobyl childhood leukemia study: background & lessons learned

**DOI:** 10.1186/1476-069X-3-12

**Published:** 2004-11-08

**Authors:** Martin C Mahoney, Kirsten B Moysich, Philip L McCarthy, Richard C McDonald, Valery F Stepanenko, Robert W Day, Arthur M Michalek

**Affiliations:** 1 Division of Cancer Prevention and Population Sciences, Roswell Park Cancer Institute, Carlton & Elm Streets, Buffalo, NY 14263 USA; 2 School of Medicine and Biomedical Sciences, State University of New York at Buffalo, Main Street, Buffalo, NY 14214 USA; 3 International Consortium for Research on the Health Effects of Radiation (ICRHER), Route 1, Bartlesville, OK 74003 USA; 4 Medical Radiological Research Center, Korolev str. 4a, Obninsk 249020, Russia; 5 Fred Hutchinson Cancer Research Center, 1733 Minor Street, Seattle, WA 98109 USA

## Abstract

Many challenges emerged during completion of a study to examine radiation dose and acute leukemia among children in areas of the former Soviet Union. In an era of globalization, our experiences might benefit others involved in multinational investigations.

## Introduction

This paper identifies the major challenges faced and the lessons learned in addressing them by the collaborative research groups involved in developing and conducting a large, multi-national case-control study of acute leukemia among children in areas of the former Soviet Union (FSU) that were most heavily exposed to radioactive fallout as a result of the April 1986 accident in reactor vessel #4 of the Chernobyl Nuclear Power Plant.

In this accident, a variety of radioisotopes including iodine (^131^I), cesium (^137^Cs, ^134^Cs), and strontium (^90^Sr), were released from the damaged reactor vessel contaminating soil, vegetation, and groundwater [1]. Fallout from the Chernobyl accident contaminated large portions of Eastern Europe, the then Union of Soviet Socialist Republics (USSR) and more distant regions. Areas of the FSU, including the now independent republics of Belarus, Russia, and Ukraine, were among the most heavily contaminated. The intent of the research project was to examine acute leukemias without specific regard to national boundaries, while recognizing the requirement to bring together investigators from these three republics in a common effort.

Acute external exposures to ionizing radiation have been etiologically linked with observed increases in the risk of all types of leukemia, except chronic lymphocytic leukemia; the risk is greatest for acute myeloid leukemia [2-12]. The association between exposure to ionizing radiation from the Chernobyl accident and the occurrence of leukemia has been summarized in a recent review [10] which highlights mixed results from published studies to date (see the review article for a comprehensive overview of published studies). Accounting for differences between the studies in the methodologies used to assess the radiological exposures, the procedures for identifying childhood malignancies and in the lengths of follow up, these authors concluded that there is not strong evidence demonstrating increases in childhood or adult leukemia from Chernobyl exposures [10]. Nonetheless, because the link between high dose ionizing radiation exposure and the development of leukemias is widely established, and children are felt to represent a uniquely susceptible population, this issue remains of high scientific and public heath interest. The study described herein focused on this most susceptible subgroup.

Collaborative studies are challenging at best; international consortia efforts are even more so. The study of border-crossing disasters such as that in Chernobyl need be investigated as a single scientific challenge to take advantage of standardizing the investigative process across national boundaries, and to afford adequate sample size and the associated statistical power to reach meaningful conclusions. This paper will not recount the events of the Chernobyl disaster or specific results of the investigation. Rather, it will share the lessons learned while conducting a collaborative, multinational study for the benefit of other investigators.

### Lesson #1: Developing a basis for collaboration

One of our first challenges was to establish an understanding of and a presence in each of the affected areas of the FSU. Multinational/multi-site studies are labor intensive and difficult to coordinate from distant locations. Therefore, the identification of a strong, on-site research team is essential. Each group must be treated as a full-member of the larger study team and be fully engaged. The local team is primarily responsible for study implementation. It is imperative that local teams with relevant expertise be formed. Our teams employed physicians, epidemiologists, statisticians, and dosimetrists. Local teams participated in study design, as well as study management in the field and troubleshooting problems. This required teams that were extremely knowledgeable about geopolitical boundaries, location of cities/small villages, governmental structures, and the health care delivery system. The latter area was particularly important given that the entire study hinged on the identification of acute leukemias and pair matched healthy controls. The health care systems in Belarus, the Russian Federation, and Ukraine have been adapted from the FSU system. Each Oblast (an Oblast is a large administrative unit – equivalent to a state in the United States) has 1–2, and in rare cases 4, large cancer treatment and diagnostic facilities (termed oncodispensaries). There are separate facilities for childhood (i.e., <16 years of age at time of diagnosis) and adult patients. In addition to providing nearly all cancer-related medical treatment, oncodispensaries house records of all reported cancer patients within the Oblast. These treatment centers contained critical source records vital to the success of our study.

On-site research teams knew how to identify cases (e.g., review of oncodispensary records), where to identify controls (e.g., polyclinics), and what agency permission was needed to obtain access to these data. This would have been impossible for an "outsider" to do. The identification of committed and skilled collaborators from each of the Republics facilitated project coordination and monitoring from outside the FSU. However, not surprisingly, there have been some difficulties related to language and culture. Although English language proficiency is progressing rapidly within the FSU, many of the individual researchers were unable to effectively communicate via English. Moreover, the collaborators based in the USA were even more limited in their Russian language fluency. This issue of language was addressed early in this collaboration through a consensus that English would be used as the common language, which was endorsed by our FSU collaborators. Certain investigators were well trained in English and the Consortium relied on simultaneous interpretation for initial meetings of collaborators, and then switched to having an interpreter present to provide general and personal assistance as needed. All printed materials, such as study protocols and survey instruments were produced in dual language versions (Russian and English) and verified via back translation.

Since the late 1990s, key project staff members in the USA and each FSU Republic maintained frequent linkages through e-mail communications at intervals between site visits and periodic project reviews. However, it should be noted that prior to the late 1990s, e-mail was scarce in the FSU and somewhat unreliable. International communications were first established via facsimile transmissions then evolved to an electronic platform (e-mail) as Internet coverage in the FSU expanded.

### Lesson #2: Infrastructure Development

**Figure 1:**
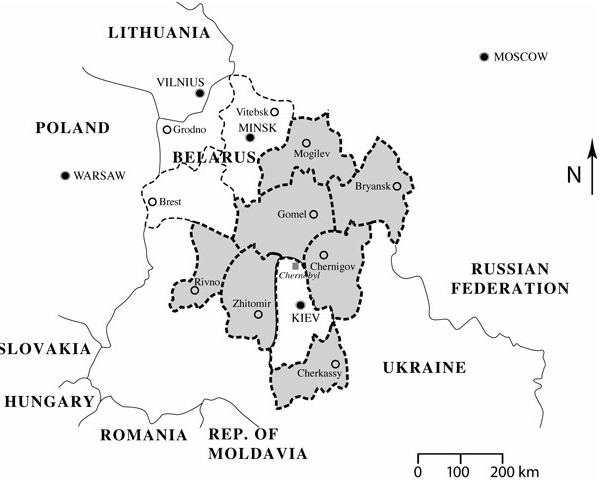
**Regions surrounding the Chernobyl Nuclear Power Plant **Shading identifies areas included in the International Consortium for Research on the Health Effects of Radiation (ICRHER) study of acute childhood leukemia: Gomel & Mogilev Oblasts in Belarus; Cherkassy, Chernigov, Rivno, & Zhitomir Oblasts in Ukraine; and Bryansk Oblast, Russian Federation. Solid lines identify boundaries between countries/republics. Shaded square in center of figure identifies location of Chernobyl Nuclear Power Plant in northern Ukraine.

This study was supported by the International Consortium for Research on the Health Effects of Radiation (ICRHER), based in the United States. (A series of articles summarizing two research conferences appears in a special supplement of the Stem Cells journal [13].) Our research objective was to examine the relationship between exposure to chronic low doses of ionizing radiation and the incidence of acute leukemias among children in the 3 FSU republics without specific regard to national boundaries (see figure 1), while recognizing the obligation to bring investigators from the three republics together with each other and those in the USA in a common effort. Individuals in the 3 republics exposed during childhood to radiation from Chernobyl were felt to represent a uniquely susceptible group and an appropriate population for a retrospective study.

The ICRHER was incorporated in June 1993. It was born of the insight and enthusiasm of one man, the late Admiral Elmo Zumwalt, Jr., United State Navy (retired), who was concerned about environmental exposures and cancer risk; particularly, how such exposures might affect military personnel. Admiral Zumwalt was also concerned about the humanitarian aspects of accidental exposure to toxic agents, including ionizing radiation. The Chernobyl reactor accident provided an opportunity to pursue both these interests.

Initially, the ICRHER (the "Consortium") was comprised of collaborative groups at institutions in the United States of America (USA) and FSU republics, specifically, the Baylor College of Medicine, Houston, Texas; the Research Center for Radiation Medicine, Kiev, Ukraine; the Fred Hutchinson Cancer Research Center (FHCRC), Seattle, Washington; the National Center for Hematology, Moscow, Russia; Hadassah Medical Organization, Jerusalem and Haifa, Israel; and the National Marrow Donor Program, Minneapolis, Minnesota. The Bridgeport Hospital/Yale University and the Research Institute of Radiation Medicine and Endocrinology, Minsk, Belarus, joined the Consortium later. The final research team for the multinational Leukemia case-control study includes the FHCRC in collaboration with the Medical Radiological Research Center in Russia and two groups from the Roswell Park Cancer Institute (one working with the Research Institute of Radiation Medicine and Endocrinology, in Minsk, Belarus and another with the National University "Kiev-Mohyla Academy", Kiev, Ukraine).

The Consortium helped to bring structure and organization in a variety of areas, from assembling the principal investigators to creating working groups to empanelling external advisory boards. A corporate entity or central office was formed to coordinate these activities, and to manage common logistic requirements, to draw up contracts for U.S. and Israeli institutions, to support the FSU collaborators, and to provide an administrative office of record. Satellite offices were established in Kiev, Minsk, Moscow, Obninsk, Bryansk and Jerusalem to provide administrative, communication and logistical support to the research activities in the FSU and Israel. FSU offices, as will be seen later, were critical to the success of this study.

Given the changes in leadership and make up of the ICRHER since its inception, it was imperative to maintain flexibility and responsiveness to change. Modifications in research partners may occur for many reasons. Therefore, it is critical that some type of "corporate entity" exist to aid in these transitions through detailed documentation and to provide continuity at the executive/advisory level.

Consortium investigators developed a pilot study to test the feasibility of initiating a full-scale epidemiologic investigation, and to determine whether needed collaborations could be established. The primary goals were to assess radiation dose, to identify individuals at risk for radiation-related illness (including émigrés to Israel), to develop data collection instruments and common study protocols, to elucidate mechanisms of radiation damage, and to establish core support through the central office. The original group invested considerable time in reviewing exposure data, the populations involved, and possible health outcomes. Following these critical discussions, a decision was made to launch a pilot investigation of acute leukemia. This study would prove challenging, but it was facilitated by the knowledge and experiences gleaned from the feasibility study.

### Lesson #3: Study Design

Based on the feasibility and pilot studies, a consensus decision was made to begin a multinational leukemia case-control study in Belarus, the Russian Federation, and the Ukraine, including the Israeli component (the Israeli studies concluded in August 2000). The focus has been on the most radiosensitive malignancy in the most radiosensitive at-risk age group: occurrence of acute leukemia in those 0 to <6 years at the time of the accident [2,10].

Initially, the aim of the Consortium was to investigate the occurrence of acute leukemia among individuals who were 0 to age 20 years at the time of the Chernobyl accident. The original population was selected due to early indications of high radiation dose exposures and easily achievable sample size estimates based on power calculations. When doses were not found to be as high as predicted, the sample size requirements became exceedingly large. Therefore, the researchers decided to narrow their focus to individuals aged 0 to <6 years at the time of the accident, since they represented a much more radiosensitive subpopulation. Hence, there is the need for well-designed feasibility studies to provide critical information in study planning and decision-making.

The final study population included 421 confirmed cases of acute leukemia and 842 population controls pair-matched to cases on age, gender, and type of residence. All participants were *in utero *to <6 years of age at the time of the Chernobyl accident in April 1986.

#### Case identification

Case identification was often difficult, particularly when compared to studies conducted in the U.S. that typically rely on either population-based cancer registries or hospital-based rapid case identification. Many regions studied did not have an up-to-date, population-based cancer registry. And, since nearly all medical records in the FSU exist in paper form, exhaustive manual reviews of hard copy records were often required. This task was somewhat simplified by the local custom of referring cancer cases to regional oncodispensaries, thereby, restricting record reviews to a finite number of facilities. Moreover, it was also necessary to review death files to ascertain cases not previously brought to medical attention. Trained physicians reviewed the records at the oncodispensaries in Belarus, the Russian Federation, and the Ukraine. Records were retrieved for all potential patients (i.e., date of birth, date of diagnosis, and residence location). After verifying eligibility, trained interviewers contacted parents of these patients and scheduled an interview.

#### Control selection

Control selection was equally vexing. In the US, researchers typically use neighborhood controls, random digit telephone dialing methods, and/or computerized databases [14-16]. In the FSU, control selection began by meeting with district health officials to obtain permission to review polyclinic records. Considerable time was then spent reading through racks of paper files and recording information for potential participants. Interviews were completed with two controls pair-matched to each case based upon age at diagnosis, sex, region/district of residence, and type of settlement.

The research teams visited the polyclinic and randomly selected 20 potential controls fitting the inclusion criteria. Interviews were generally scheduled and completed for the first two potential controls on the list. Other names on the list were contacted and scheduled for interviews as needed. Over 90% of potential controls contacted by the research teams agreed to participate. These high rates of case and control participation may be attributed to the dedication and resourcefulness and commitment of the research teams and the use of modest incentives (food baskets).

#### Interview

As formidable as the above tasks were, they paled in comparison to actual data collection. During the feasibility study, investigator meetings were held in the US to develop common data collection instruments. These instruments were structured to address issues of cultural and language compatibility for use in the three Republics. While Russian was adopted as the standard language for data collection, study instruments contained both Russian and English text. Instruments were back translated into English to ensure accuracy of translation.

Next, a team of interviewers needed to be recruited and trained. We relied almost exclusively on recruiting physicians who were familiar with the disease process, were credible representatives, were respected by study participants, and who could be depended on to provide accurate and verifiable data. The mix of urban areas and rural settlements presented logistical challenges in terms of tracking participants, arranging appointments, and completing face-to-face interviews. To overcome this challenge, some interviewers mailed letters to introduce the project, and to request that the participant contact the interviewer. Others traveled to local communities to personally discuss participation and to schedule interviews. Still others used local contacts to identify participant places of employment for either telephone or direct contact.

Traveling to interview study participants was another challenge. In contrast to conducting research in the US where there are widespread and reliable telecommunication systems, well-developed highway systems, and accurate maps, FSU travel was more problematic. For example, not all roads to small villages in the FSU are paved and most are limited to a single lane in either direction. Roads can be particularly treacherous in the winter and rainy seasons. Maps do not always show the precise location of small villages. Villages have neither street signs nor house numbers. Further, the collapse of the Soviet Union resulted in changes in the names of many streets, settlements, and villages. This arduous process of locating and interviewing controls took considerable effort and time. Where interviewers in the U.S. are generally out and back in the same day, FSU colleagues were out in the field for days at a time and would be fortunate if they could complete a handful of interviews each day. These differences in operations must also be factored in to the overall cost of the study. Finally, local residents tended to be wary of strangers and special introductions were often necessary to gain entry.

One cannot overstate the importance of having a well-trained and dedicated on-site research team that is familiar with cultural norms and local "maps". The two-hour, face-to-face interviews were generally completed with the mothers of cases and controls. Interview items were developed by US and FSU scientists and included sections addressing demographics, general health status, maternal and paternal occupational history, and a detailed questionnaire for obtaining information necessary for developing estimates of individualized internal and external radiation exposures, including questions regarding consumption of locally produced milk, meats, and vegetables; residence history and type of housing structure; use of protective measures immediately after the accident; and time spent outdoors.

### Lesson #4: Working Groups

Those involved in multinational or multi-site investigations may consider the creation of Working Groups to monitor the various components of the study. As has been utilized in other international studies [17], the lead investigators could separate the overall study into its basic components and organize working groups that represent all collaborating teams. For example, we established a leukemia diagnostic working group comprised of hematologic morphologists and hematologists from representative ICRHER institutions, and chaired by a leading pediatric hematologist who was not associated with the Consortium. Members completed blinded reviews of bone marrow pathology slides, or other information (e.g., clinical histories, laboratory data) for cases without slides, and then assigned a histopathologic diagnosis based upon group consensus. A subset of cases was randomly selected for repeat review to assess consistency. Results of this review process affirmed the accuracy of acute leukemia diagnoses made within these areas of the FSU [18].

A common methodology to assess individual absorbed radiation doses and corresponding uncertainties was developed and tested by the dosimetry intercomparison working group (DIWG) for all subjects based on interview data and available exposure data. The interview collected detailed exposure data for each subject from the time of the Chernobyl accident until the reference date. It should be noted that interview data alone were not sufficient to determine individual dose estimations. For retrospective dose estimation specialized radioecological data were necessary. These resources provided information concerning local soil types, food contamination with different radionuclides, dates of radioactive cloud arrivals to each local community, etcetera. These data were collected by the members of DIWG based on information published within the FSU.

Dosimetrists are a good example of the specialized personnel that need to be a full-time, on-site presence. They have critical knowledge of the local area as well as access to primary data essential in quantifying exposure. Individual dose estimates reflected local conditions (e.g., contamination levels, soil type, soil to milk transfer coefficients) at each location where a particular subject lived during the appropriate time interval. The subject's residence history and other important personal information, such as milk consumption and food sources, were collected during the standardized interviews by trained examiners. Fieldwork was performed jointly by physicians and dosimetrists.

A Data Analysis Working Group was essential for oversight of the analytic phase. Composition included representatives from each site with a strong chair to maintain focus and momentum. Also, a Data Audit Working Group was established at study inception to conduct periodic reviews to assure adherence to study protocols. And finally, a centralized Data Coordination Office served as a repository of the common dataset and oversaw periodic computerized checks for quality and completeness.

## Conclusions

Our experiences in the organization and successful implementation of a multinational, retrospective study of acute leukemia in regions impacted by the Chernobyl disaster have been highlighted. Issues identified during the implementation of our multi-national epidemiology study, along with strategies for resolution are summarized in table 1. While trained research teams within each Republic were responsible for collecting data, we relied on a distinctive series of working groups of collaborators from participating institutions to coordinate various aspects of the study such as case confirmation, data quality, dosimetry, and data analyses. This allowed all project teams to remain interconnected and equally involved while utilizing the unique expertise of various collaborators.

**Table 1:** Potential issues regarding the implementation of multi-national epidemiology studies

**Challenge:**	**Resolution strategy:**
•Language	•Dual language versions (Russian & English) for all printed materials; use of interpreters
•Geographic distance between collaborators	•e-mail accounts for key collaborators; site visits, progress meetings
•Limited experience with epidemiology	•Mandatory training workshops for interviewers; audits to assure compliance with protocols
•Subject ascertainment	•Cases identified through manual record reviews at oncodispensaries and childhood oncology centers; controls identified from manual review of raion medical records
•Limited comprehensive cancer registry data	•Manual records review at oncodispensaries, childhood cancer centers and mortality files
•Lack of telephone to contact participants	•Mailed letters of introduction; field trips to communities
•Locations of study participants	•Field trips for data collection; assistance of local residents
•Radiologic contamination data in multiple locations	•Visits to multiple Institutes & offices; contacts of collaborators
•Adequate communications	•All research sites provided immediate Internet access
•Timely compensation for local investigators	•Direct pay facilitated by USA agencies (e.g., Civilian Research and Development Foundation)
•Common research protocols and joint methodology for individual radiation assessment	•Periodic meetings of all USA/FSU investigators to promote personal relationships and scientific value of combined data
•Data collation and analysis	•Establish Data Coordination Office in the Former Soviet Union •Transfer all data electronically
•Data access and archives	•Access by mutually-agreeable policy •Transfer data to USA institution for permanent storage
•Multidisciplinary international study	•Highly cooperative, joint international consortium with working groups

The breakup of the former Soviet Union in 1991 created national autonomy in Belarus, the Russian Federation, and Ukraine. Although the study area for this project included selected regions of the FSU, the intent was to study acute leukemia without regard to specific national boundaries, while recognizing the requirement to bring together investigators from these three Republics in a common effort. Proprietary concerns and country-specific restrictions on the sharing of scientific information were thoroughly addressed to gain agreement and to facilitate the pooling of analytic information.

The Consortium played an integral role in providing infrastructure support for this project through the appointment of project support administrators at each research site to oversee communications, equipment procurement/maintenance, and compensation. The fiscal aspects of supporting research are unique to each country. A careful examination of collaborating scientific institutions and the financial regulations of each participating country prior to setting up any support mechanisms is critical, and may result in country-specific arrangements. Essential computer, laboratory, and communication equipment was supplied. Computer software, which was compatible across the three research settings, was installed and upgraded periodically. Equipment was segregated and secured to insure exclusive use by project staff.

It should be emphasized that this investigation represents the largest retrospective study examining the relationship between Chernobyl radiation exposures and risk of acute leukemia conducted to date and the only research effort to bring together data from the most exposed areas into a single study; results of the multinational case-control study are presented in a separate paper [19]. The conduct of multinational epidemiologic studies presents numerous challenges [17,20], including issues such as language, physical infrastructure, telephone coverage, and road conditions, as well as geographic distances and issues of participant ascertainment. However, as demonstrated by our experiences, these challenges can be effectively overcome through attention to organization, communication, and quality assurance. Moreover, these challenges are greatly offset by unique opportunities to yield information of great significance to science and society.

## List of Abbreviations

FSU, former Soviet Union

USSR, Union of Soviet Socialist Republics

USA, United States of America

ICRHER, International Consortium for Research on the Health Effects of Radiation; the "Consortium"

FHCRC, Fred Hutchinson Cancer Research Center

DIWG, dosimetry intercomparison working group

## Competing Interests

The author(s) declare that they have no competing interests.

## Author contributions

MCM and AMM were responsible for the study concept. MCM and AMM drafted the manuscript; KMM, PLM, RCM, VFS and RWD provided critical review and input. MCM, KMM, PLM, RCM, VFS, RWD and AMM participated in interpretation, as well as in data acquisition efforts. All authors read and approved this manuscript.

## Pre-publication history

The pre-publication history for this paper can be accessed here:


